# Supplementary material for: Chromothripsis during telomere crisis is independent of NHEJ, and consistent with a replicative origin
Source: Genome Res. 2019 May;29(5):737–49. doi: 10.1101/gr.240705.118 (PMC6499312; doi:10.1101/gr.240705.118)
Supplement: Supplemental Material [file supp_gr.240705.118_Supplemental_file_1.zip › contigs/annotated_contigs/DB88/contig.2.DB88_length_710_mean_cov_11.7676056338.docx]

**DB88_length_710_mean_cov_11.7676056338**

```
AAACAATTTTGTTTGTGTGTAGACAAAAACCACTTTTTCATATAAATGTCTAAGCATATTTTTCTCAACTTATTCTACTAAAATTTAAG
 >chr7:142130378-142130692 - E=4e-174 p=4e-02
TTTGGATTTTCACCACTAAGAATAAATGTACCTGAAATACATCTCATGTAAGAAATAAGATACAGACCTAGGAATGGGAGGGAAGGACA

TATATTCAGTGAAGAAACAATAGCTTGTCAAAAAGAGCCCTAAGTGGACACCTGGAGCTGACGGTGGAGAGGTAGGTGGAGGGCAAATG

AGCATGAACTTTGTCAGTCTATTGTCTTGTAGAAGAAAGACAGCCAG|TGTGGCC|AGTGTGGCCACTCTCCATCTCAGTCCCTGGAAG
                                                       >chr7:142116990-142117319 - E=7e
TTGTCCAGAGTTCCCTGAGAATCCCTGCTCTGGGGCAGAATCACCAAGGCTCATCCTCACCAGCTCCCCACAGGCTCCAGCAGGGCTTT
-158
CCTGCCAGGTGTAGGGCACAGGAATGGCTCTGTCTGTCTCTCAGGTAGAGGGAGGCCAAACAGTGATGTTTGTATAAGAGGGACTGGGT

TTCTGTGTCTCCATTAAAGTATTTGACAATATTTTCTTTGTTTTTTTTTTTTTGGTTTATTTTTCACTCTCGCCAATGAGGAGAGACAT

CCTTTGGTATTTATTTTGTTTTTGGC|CATTGCGACGGTTTTTGGGGCTGCGTGAAATTCAGAAATTGTACATTTTTTTTCTTTTCAGC

C
```
